# Polyphasic linkage and the impact of ligand binding on the regulation of biomolecular condensates

**DOI:** 10.1063/5.0050059

**Published:** 2021-06-15

**Authors:** Kiersten M. Ruff, Furqan Dar, Rohit V. Pappu

**Affiliations:** 1 Department of Biomedical Engineering and Center for Science and Engineering of Living Systems (CSELS), Washington University in St. Louis, St. Louis, Missouri 63130, USA; 2 Department of Physics, Washington University in St. Louis, St. Louis, Missouri 63130, USA

## Abstract

Cellular matter can be spatially and temporally organized into membraneless biomolecular condensates. The current thinking is that these condensates form and dissolve via phase transitions driven by one or more condensate-specific multivalent macromolecules known as scaffolds. Cells likely regulate condensate formation and dissolution by exerting control over the concentrations of regulatory molecules, which we refer to as ligands. Wyman and Gill introduced the framework of *polyphasic linkage* to explain how ligands can exert thermodynamic control over phase transitions. This review focuses on describing the concepts of polyphasic linkage and the relevance of such a mechanism for controlling condensate formation and dissolution. We describe how ligand-mediated control over scaffold phase behavior can be quantified experimentally. Further, we build on recent studies to highlight features of ligands that make them suppressors vs drivers of phase separation. Finally, we highlight areas where advances are needed to further understand ligand-mediated control of condensates in complex cellular environments. These advances include understanding the effects of networks of ligands on condensate behavior and how ligands modulate phase transitions controlled by different combinations of homotypic and heterotypic interactions among scaffold macromolecules. Insights gained from the application of polyphasic linkage concepts should be useful for designing novel pharmaceutical ligands to regulate condensates.

## I. INTRODUCTION

Membraneless biomolecular condensates provide spatial and temporal organization over cellular matter, specifically proteins and nucleic acids.^1–4^ Condensates form in response to different stimuli that activate or repress gene expression, translation, transcription, protein degradation, or epigenetic modifications.^1,5–11^ Likewise, the dissolution of condensates involves a combination of active and regulatory cellular processes.^12^ Spontaneous phase transitions are driven by key, condensate-specific, multivalent molecules that are known as scaffolds.^1^ Scaffold molecules are required for condensate formation.^13,14^ This implies that a knockout of a scaffold molecule must lead to the abolishment or large-scale disruption of condensate formation.^15^ Recent studies have shown that condensates such as nucleoli, nuclear speckles, germline granules, signaling bodies, processing bodies (P-bodies), and stress granules form and dissolve via reversible phase transitions of condensate-specific scaffolds, which can be multivalent proteins, nucleic acids, or both.^8,13,14,16–19^ Are there ways for cells to exert thermodynamic control over phase transitions driven by scaffolds? This question, which is the focus of the current review, brings us to the topic of *polyphasic linkage,* a theory formulated by Wyman and Gill that explains how binding and linkage relations lead to control over phase transitions through ligand binding.^20^ The concept of polyphasic linkage, formalized by Wyman and Gill, arose, in part, from the work of Rupley, who developed a framework for understanding how changes to protein solubility are affected by small molecules that enable protein crystallization.^21^

Scaffolds are biological instantiations of associative polymers, defined by a multivalence of attractive groups known as *stickers.*^22–26^ Associative polymers can undergo two types of phase transitions, *viz*., phase separation and percolation.^23,24,26,27^ Linear or structural interaction motifs within scaffolds are the stickers that form reversible physical crosslinks with one another.^28,29^ These crosslinks involve a combination of short- and long-range interactions that span a range of interaction strengths.^28,30,31^ The overall valence of stickers is determined by the total number of stickers and the coordination number per sticker.^29^ Reversible crosslinks among stickers give rise to connected networks of scaffolds. Above a threshold concentration known as the percolation threshold, *c*_perc_, the network of crosslinks becomes system spanning, and the underlying transition is known as *percolation.*^27,29^

The interplay of polymer–polymer and polymer–solvent interactions controls a second type of transition, known as *phase separation*. This is a density transition, whereby a polymer solvent mixture, under the appropriate solution conditions, separates to form a dilute polymer-deficient phase and a dense, polymer-rich phase that coexist with one another.^27,32,33^ The concentration threshold above which phase separation occurs is denoted as *c*_sat_, and the concentrations in the coexisting dilute and dense phases are denoted as *c*_dil_ and *c*_den_. Note that *c*_sat_ and *c*_dil_ are the same for a binary mixture of polymer plus solvent. For associative polymers, it is often the case that *c*_den_ is greater than *c*_perc._^23,28^ Accordingly, the associative polymers form a droplet-spanning percolated network, and the timescales associated with the making and breaking of crosslinks will determine the timescales over which the condensate behaves like an elastic vs viscous material.^22,29^ As a result, condensates that form via *phase separation–aided percolation* transitions are best described as networked viscoelastic materials.^34^

**FIG. 1.**
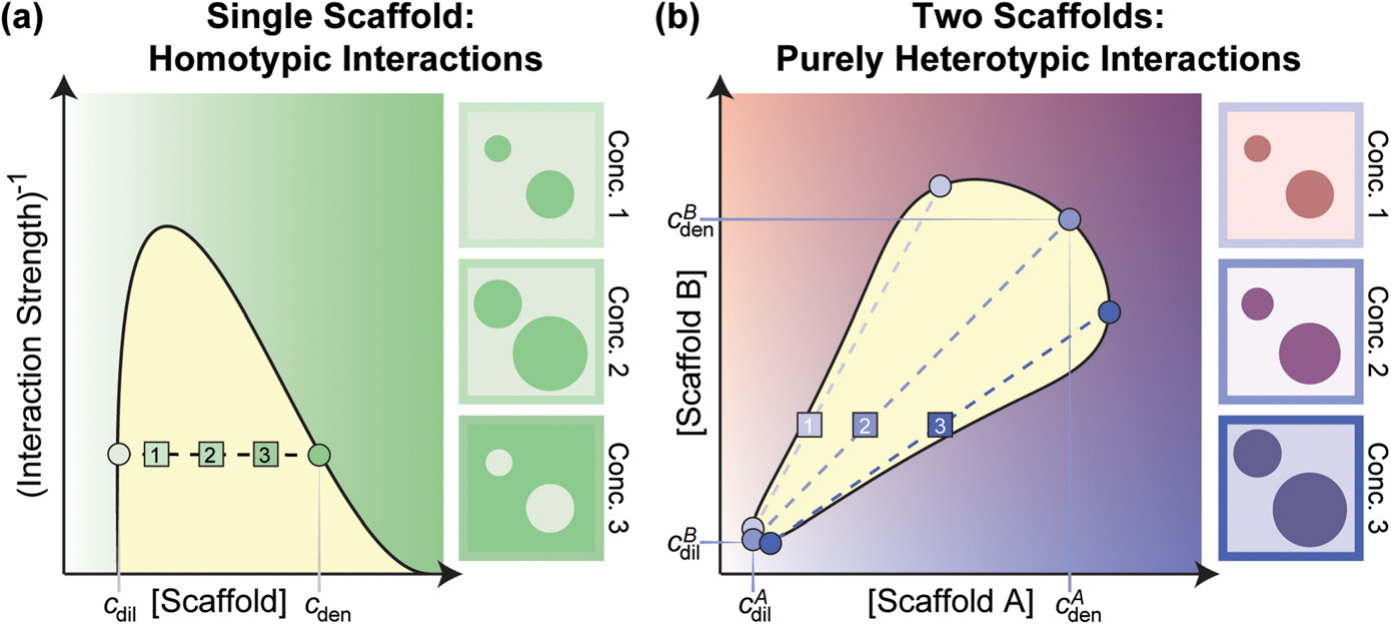
Phase boundaries for a single scaffold system driven purely by homotypic interactions and a system comprising of two co-scaffolds where phase separation is driven purely by heterotypic interactions. (a) For the single scaffold system, the scaffold phase behavior can be described as a function of scaffold concentration and some measure of interaction strength, usually temperature. The binodal (black line) denotes the boundary between the one-phase (green region) and two-phase (yellow region) regimes. For points within the binodal, the system separates into two co-existing phases: a dilute (dil) scaffold-poor phase and a dense (den) scaffold-rich phase. The concentration of the scaffold in the dilute phase (*c*_dil_) is given by where the tie line (dashed line) crosses the left arm of the binodal and the concentration of the scaffold in the dense phase (*c*_den_) is given by where the tie line crosses the right arm of the binodal. When phase diagrams are drawn as a function of temperature on the y-axis, the tie line is horizontal. This implies that at a given temperature, regardless of the input scaffold concentration (1, 2, or 3), the system separates to the same *c*_dil_ and *c*_den_ values. However, the volume of the dense phase increases as the input scaffold concentration increases. It is important to remember that the tie lines are not necessarily horizontal if interaction strength is modulated by changing pH or salt concentration. (b) For the system with two co-scaffolds, the phase behavior for a given set of solution conditions is given by a function of the concentration of scaffold A (blue) and the concentration of scaffold B (red). For purely heterotypic interactions, the binodal is a closed loop, where *c^A^*_dil_ and *c^B^*_dil_ denote the scaffold A and B concentrations in the dilute phase and *c^A^*_den_ and *c^B^*_den_ denote the scaffold A and B concentrations in the dense phase. Tie lines (dashed lines) show the corresponding dilute and dense phase scaffold concentrations associated with three representative input concentration conditions (1, 2, and 3). In both (a) and (b), the boxes to the right of the phase diagrams show the relative size and composition of the two phases given an input concentration condition of 1, 2, or 3. Lighter colors denote lower concentrations and darker colors denote higher concentrations.

If only one type of multivalent macromolecule (hereafter referred to as a scaffold^1^) is necessary and sufficient to drive condensate formation, then the interactions that drive phase transitions are effectively homotypic in nature. In a plane defined by scaffold concentration and interaction strength, a phase boundary or *binodal* delineates the region where phase separation is realized [Fig. 1(a)]. Phase separation can be controlled by changes to temperature, pressure, pH, salt, or other excipients.^35–39^ When more than one type of scaffold is necessary for driving phase separation, then a blend of homotypic and heterotypic interactions or purely heterotypic interactions will be involved.^8,40^ In such cases, for a fixed set of solution conditions, the phase boundary is constructed in the hyperplane defined by the concentrations of all the relevant scaffold molecules. A schematic of such a phase boundary is shown in Fig. 1(b) for the case where two scaffold molecules drive phase separation. In this case, the concentrations of scaffolds A and B in the dilute and coexisting dense phase will change with the input stoichiometric ratio of the two types of molecules.

Condensates typically contain hundreds of distinct types of macromolecules, and yet only a handful of macromolecules appear to be scaffolds that drive condensate formation.^15,41,42^ This implies that a vast majority of the molecules that makeup condensates are non-scaffold molecules.^13,43^ Some of the non-scaffold molecules are *ligands* that preferentially bind to scaffold molecules across the phase boundary.^20,44^ *Preferential binding* quantifies the relative affinity of ligands to sites on scaffold molecules within the dense vs dilute phase. Specifically, phase separation is weakened if the ligand prefers to bind to sites on the scaffold in the dilute phase vs the dense phase. Conversely, phase separation is strengthened if the ligand prefers to bind to scaffold sites in the dense phase when compared to scaffold sites in the dilute phase. The extent to which phase separation is weakened or enhanced by specific types of ligands can be put on a quantitative footing using the framework of polyphasic linkage.^20,44,45^

## II. POLYPHASIC LINKAGE

The establishment of a phase boundary and the emergence of two or more coexisting phases arises from a thermodynamic instability in the one-phase system. In this situation, the overall free energy is minimized by setting up coexisting phases. In the case of two coexisting phases, there is one phase boundary, and the compositions and densities of the phases are governed by conditions for chemical and mechanical equilibria across the phase boundary. Chemical equilibrium is achieved by equalizing the chemical potentials of all components across the phase boundary.^1,46,47^ Equalizing the chemical potential of the macromolecular scaffold in the presence of a ligand (denoted as L), leads to an expression for *c*_dil_ that is written as follows: $c_{\text{dil},L}=c_{\text{dil}}\left(\frac{P_{\text{dil}}}{P_{\text{den}}}\right)$.^20^ Here, *c*_dil,L_ is the concentration of the macromolecular scaffold in its coexisting dilute phase, measured in the presence of the ligand L whereas *P*_dil_ and *P*_den_ are the binding polynomials that describe the binding of the ligand to the sites on the scaffold in its dilute vs dense phase, respectively. A ligand can interact with one or more sites on the scaffold in either the dilute or dense phase. If we assume that the ligand binds scaffold sites independently within each of the phases, then the binding polynomials are written as: $P_{X}=1+\sum_{i=1}^{n_{\text{b,X}}}\beta_{i}^{\left(X\right)}a_{L,X}^{i}$. Here, X is either dil or den depending on whether we are referring to the dilute or dense phase, respectively; *n_b_*,_X_ is the number of scaffold sites available to the ligand in phase X; the parameters $\beta_{i}^{\left(X\right)}$ denote the *i*th order binding constants in phase X; and *a*_L,X_ refers to the activity of the ligand, which is the concentration of free ligand in phase X.

**FIG. 2.**
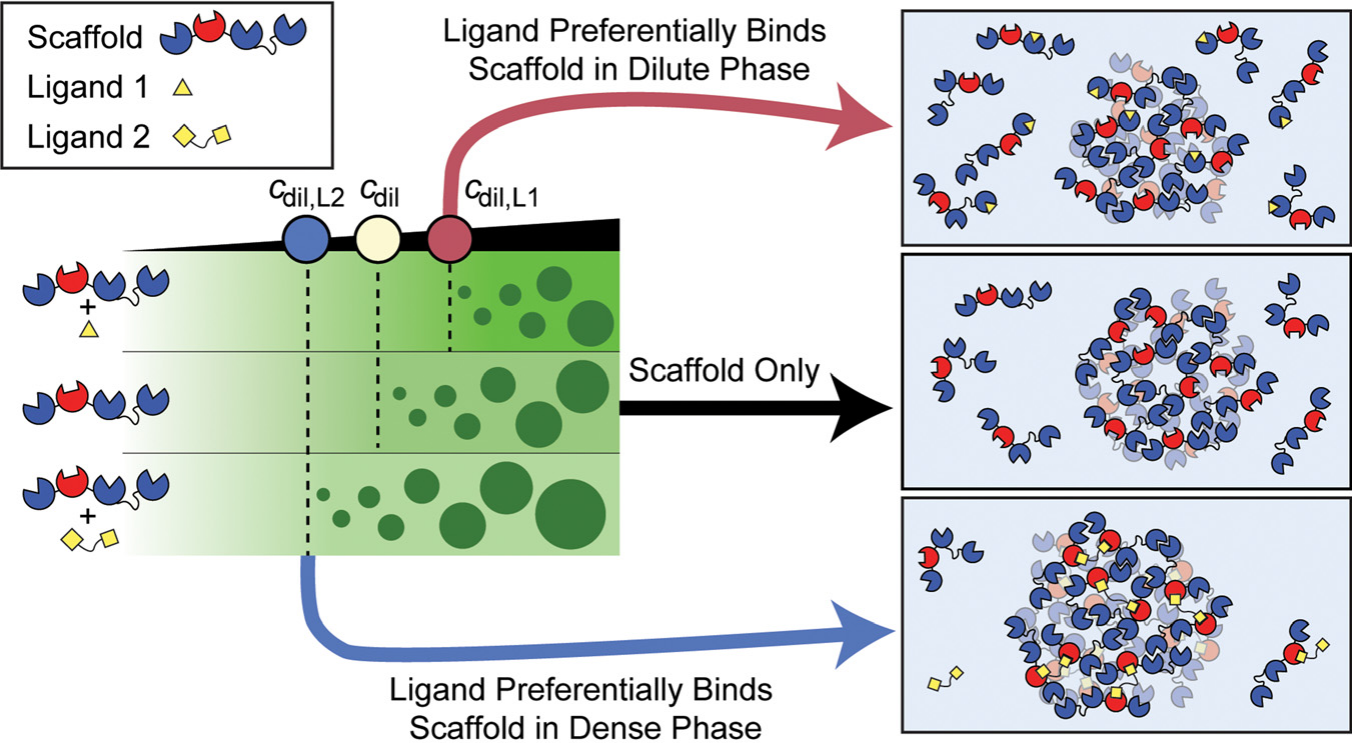
Schematic of polyphasic linkage. Ligands that bind preferentially to sites on the scaffold in the dilute phase (ligand 1) suppress phase separation by increasing *c*_dil_. In contrast, ligands that preferentially bind the scaffold in the dense phase (ligand 2) promote scaffold phase separation by decreasing *c*_dil_. Here, the black triangle denotes the input scaffold concentration, and the blue Pac-Men represent stickers within the scaffold molecule that drive phase separation.

The ligand is a passive client if it binds equivalently to sites on scaffolds in the two coexisting phases, i.e., if *P*_dil_ = *P*_den_. This would imply that the sites to which the ligand binds are equivalent in identity and affinity across the phase boundary. The ligand is a suppressor of phase separation if *P*_dil_ > *P*_den_, implying that *c*_dil,L_ > *c*_dil_. This happens when the overall affinity of the ligand is stronger for sites on the scaffold in its dilute phase when compared to sites on the scaffold in its dense phase. Conversely, the ligand is a driver of phase separation if *P*_dil_ < *P*_den_, implying that *c*_dil,L_ < *c*_dil_ because the ligand binds preferentially to scaffold sites in the dense vs the dilute phase. Whether a ligand is a passive client, or a suppressor vs driver of phase separation can be discerned by measuring and comparing the values of *c*_dil_ and *c*_dil,L_ (Fig. 2).

## III. RELEVANCE OF POLYPHASIC LINKAGE

Through their preferential binding effects, ligands can weaken or enhance the driving forces for phase separation of scaffold molecules. Accordingly, one way to regulate phase separation in cells is to have the saturation concentration of scaffolds be above the endogenous levels of these molecules within cells. The expression of a suitable driver ligand will lower the saturation concentration of the scaffold, thereby enabling phase separation.^48^ This type of ligand-mediated condensate formation also works in the opposite direction, whereby the expression of a suppressor ligand can destabilize and dissolve a pre-formed condensate.^49,50^ The presence of diverse arrays of ligands, whose concentrations and binding activities might regulate condensate formation or dissolution, suggests that multiple cellular knobs—in the form of ligand concentrations—can be turned to enable ligand-responsive condensate formation and dissolution.

## IV. MEASURING THE EFFECTS OF LIGANDS ON SCAFFOLD PHASE SEPARATION

**FIG. 3.**
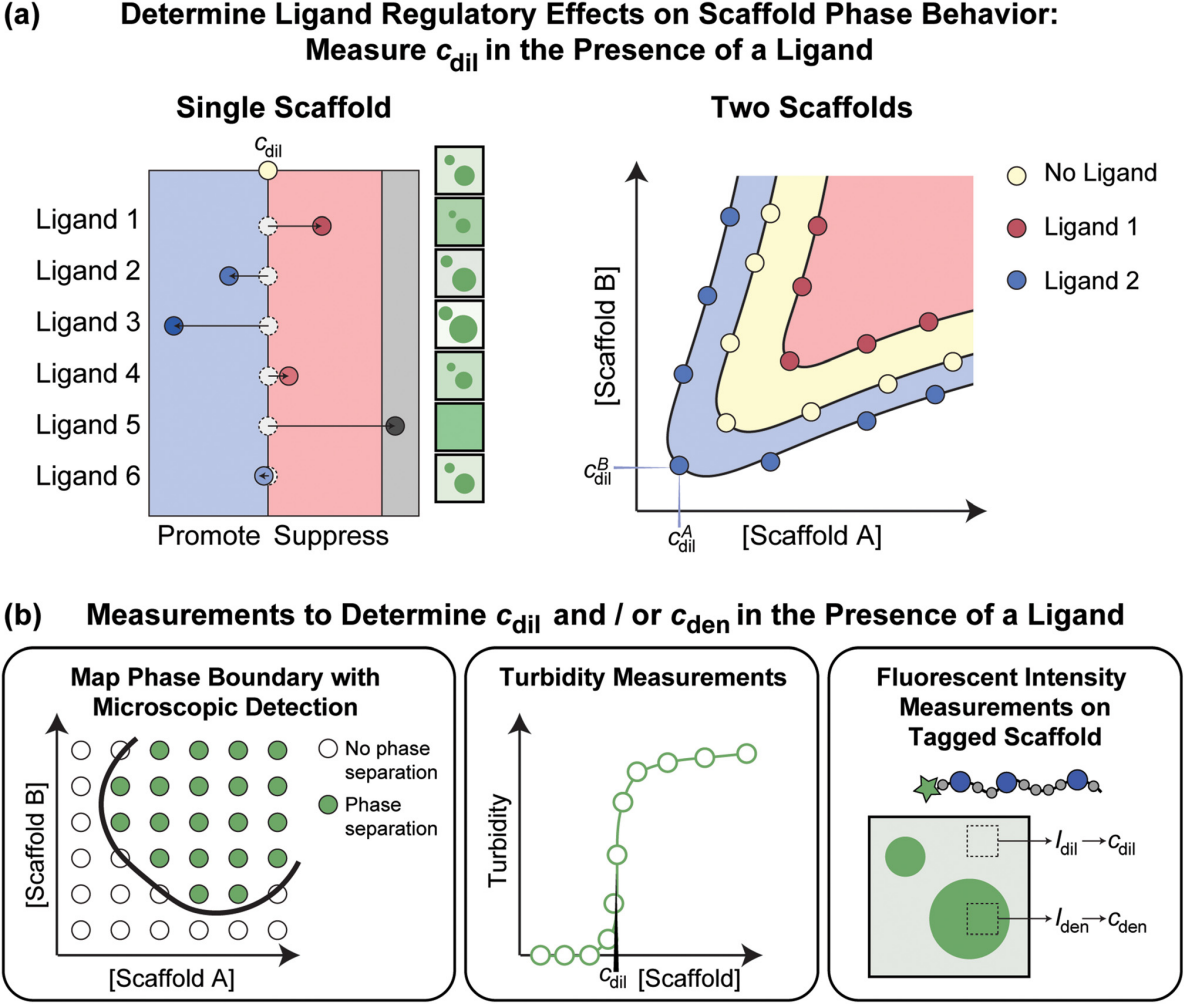
Measuring how ligands affect scaffold phase behavior. (a) To quantify how a ligand affects scaffold phase behavior, we need to measure the scaffold concentration in the dilute and dense phases in the absence and presence of the ligand of interest. If *c*_dil_ increases, then it implies that the ligand suppresses scaffold phase separation. If *c*_dil_ decreases, then it implies that the ligand promotes scaffold phase separation. (b) Different types of measurements can be deployed to measure *c*_dil_ and/or *c*_den_ in the absence and presence of a ligand. Schematics shown here depict the use of microscopy, measurements of turbidity, and measurements of fluorescence intensities within and outside condensates. The latter takes advantage of fluorescently labeled scaffold molecules.

For scaffolds that drive phase separation through homotypic interactions, the driving forces for phase separation for a given set of experimental conditions are quantified by measuring the coexisting concentrations of the scaffold in the dilute (*c*_dil_) and dense (*c*_den_) phases. Measuring how *c*_dil_ and *c*_den_ are impacted as a function of ligand concentration for different ligand types provides a direct readout of how ligands influence the phase separation of scaffold molecules [Fig. 3(a)].^44^

A variety of methods can be brought to bear on measuring the desired concentration thresholds in the absence and presence of ligands.^28,31,51–53^ Microscopy provides a yes/no answer for whether phase separation is realizable at a given set of solution conditions and concentrations [Fig. 3(b)].^8,15,54^ To assess the regulatory effects of a ligand, the two concentrations to titrate are that of the scaffold and the ligand of interest. Turbidity measurements, performed as a function of scaffold concentration, in the presence vs absence of ligands, provide a quantitative assessment of *c*_dil_ [Fig. 3(b)].^28,31,37,52,55^ However, this method is sensitive to many types of assemblies, and therefore it should be supplemented with microscopy to assess condensate formation.^51,52^

Fluorescently labeled scaffolds can be used to measure *c*_dil_ and *c*_den_ *in vitro* or in cells. Here, standard curves help with the conversion of fluorescence intensities within dilute and dense phases into estimates of *c*_dil_ and *c*_den_, respectively [Fig. 3(b)].^14,31,56^ Being able to monitor how phase boundaries shift as the expression levels of ligands are altered in cells will be important for understanding biologically relevant condensate regulation. In this context, optogenetics tools,^57^ whereby phase separation is activated by blue light and condensate formation is monitored using fluorescently labeled molecules, provide a tunable way to assess in-cell phase separation.^17,40,58^ Here, light activation effectively increases the valence of the scaffold by allowing light-sensitive domains to oligomerize. As these methods become widely used and readily deployable across different cell lines, it will be feasible to investigate how networks of ligands impact the phase behaviors of various types of macromolecules that serve as scaffolds for different types of condensates.

Centrifugation has also been utilized to determine scaffold concentrations in the dilute and dense phases in the absence of ligand.^28,31,53^ However, because this method depends on an absorbance or fluorescence signal to measure concentrations it requires that the scaffold have a distinct signal from the ligand. Otherwise, the measured concentration would be a convolution of both the scaffold and ligand concentrations. The use of microfluidics methods to measure *c*_dil_ and *c*_den_ is in its early stages,^59^ although impressive advances have been made recently.^60,61^ These methods provide the benefits of using smaller volumes, thereby increasing the number of samples that can be assessed independently.^59,62^ No matter the methods one uses, the goal is to measure *c*_dil_ and *c*_den_ of scaffold molecules *in vitro* or in cells, in the absence and in the presence of the ligand of interest.

Often it is easier to measure *c*_dil_, compared to *c*_den_, using colorimetry, turbidity, or spectrophotometry methods. Additionally, the partition coefficient (*PC*) of the scaffold molecule in relative quantities is readily accessed using fluorescent measurements. *PC*s are defined as the concentration of the molecule in the dense phase divided by the concentration of the molecule in the coexisting dilute phase.^13^ One can extract an estimate for *c*_den_ from knowledge of *PC* and *c*_dil_, determined from a complementary method, because *PC* = (*c*_den_/*c*_dil_). For a scaffold, whose phase behavior is governed by homotypic interactions, the value of *PC* should stay fixed and not change with changes to the total concentration of the scaffold. Although the scaffold *PC* is a function of the two thermodynamically relevant quantities that describe scaffold phase behavior, it is worth noting that the scaffold *PC* value itself is not unique, and any errors in the measurements of individual concentrations will be compounded in this value. Thus, reporting scaffold *PC*s along with the actual concentrations of the scaffold in the dilute and dense phase will yield a more complete description of the scaffold phase behavior.

*PC*s can also be quantified for ligands. This provides a route for profiling the complex compositions of condensates.^7,13–15,17,63^ However, recent studies have shown that the *PC*s of ligands are dependent on more than just the relative scaffold concentrations in the two phases.^43,45^ Specifically, the ligand *PC* depends on the affinity of the ligand for the scaffold and the total ligand concentration. Accordingly, the *PC* of a ligand lacks a one-to-one mapping to the scaffold *c*_dil_. Therefore, it is the effects of ligand concentrations on *c*_dil_ and *c*_den_ of scaffolds that proves to be informative rather than the *PC*s of ligands, which even though readily accessible, are uninformative regarding the effects of ligands on scaffold phase behavior.

For condensates that are formed by heterotypic interactions of *n* scaffolds, the phase behavior is defined by an *n*-dimensional phase diagram with the phase boundary defined by the concentrations of each of the scaffolds in the dilute and dense phases.^40^ According to the Gibbs phase rule, for fixed temperature and pressure, such systems can form a maximum of *n* different coexisting phases. This creates additional complexities to the problem of discerning how ligands impact phase boundaries.^29^ An example of a phase boundary for a system with two scaffolds that drive phase separation through heterotypic interactions is shown in Fig. 1(b). For such systems, the effects of ligands on phase separation are determined by measuring the dilute and dense phase concentrations of both scaffolds in the presence of the ligand [Fig. 3(a)]. Yang *et al.*^15^ used this approach to determine the effect of stress granule components on the phase behavior of G3BP1/2 and RNA, the two main scaffolds of stress granules.^15^ Ghosh *et al.* similarly measured how the phase behavior of a poly-SH_3_:poly-PRM system was regulated by different ligands.^43^ They showed that ligands can generally be classified into three types of regulators: direct interaction promotors, direct interaction suppressors, and volume-exclusion promotors. In Sec. V, we summarize the set of rules that have emerged from recent studies that help identify the features of ligands that contribute to their ability to suppress or promote scaffold phase separation.

## V. FEATURES OF LIGANDS THAT ARE IMPORTANT FOR CONTROL OF SCAFFOLD PHASE BEHAVIOR

**FIG. 4.**
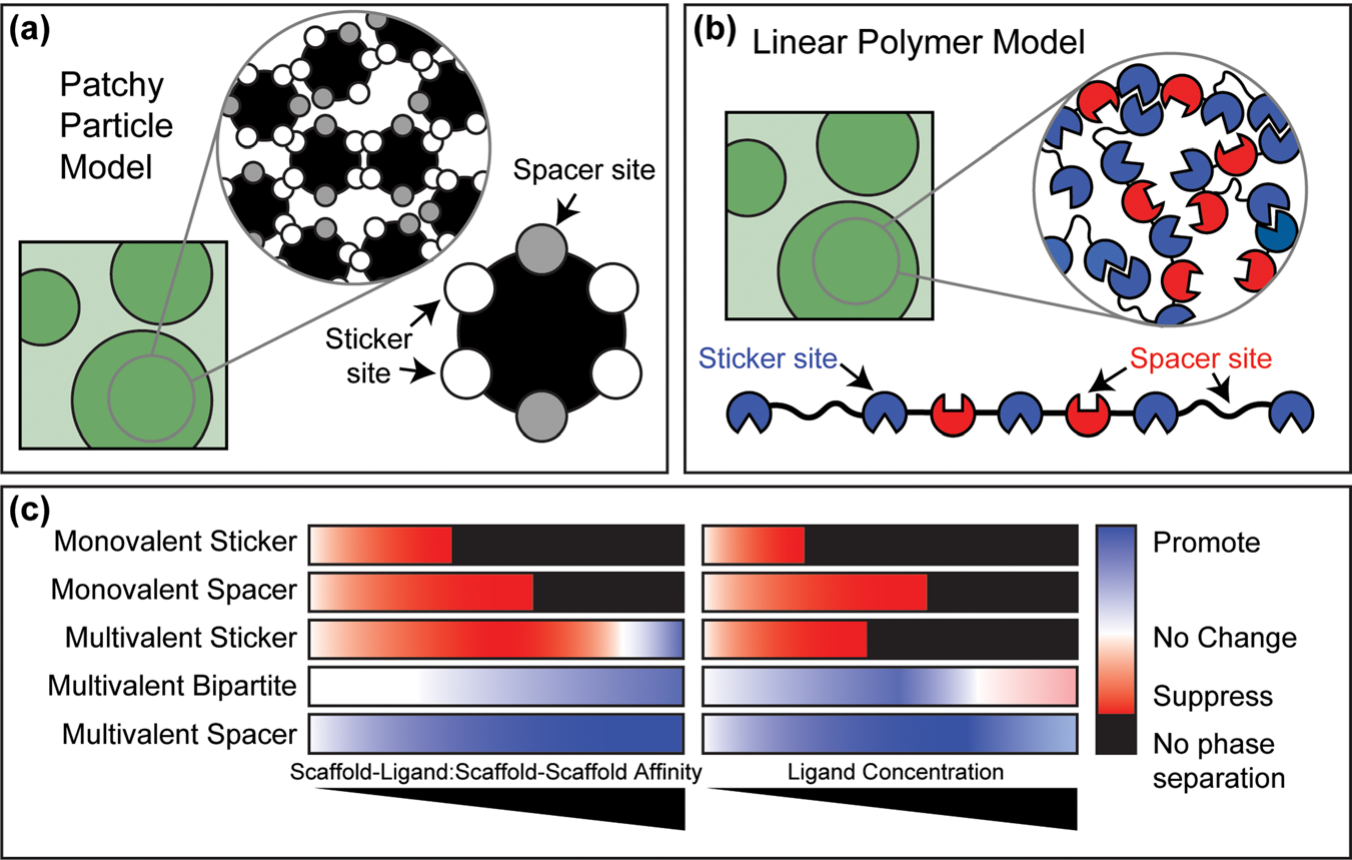
Coarse-grained models have yielded predictive rules regarding the features of ligands that suppress or promote scaffold phase behavior. (a)–(b) Schematic of the patchy particle and linear polymer models used for modeling ligand effects on scaffold phase behavior. (c) The general effects on scaffold phase behavior for five different ligand types as a function of relative scaffold–ligand to scaffold–scaffold affinity or ligand concentration.^43,45,64,65^ Ligands are designated as follows: Ligands that are monovalent and that bind to sticker vs spacer sites on scaffolds are designated as *monovalent sticker* and *monovalent spacer*, respectively. A multivalent ligand that binds to sticker sites on scaffolds is designated as *multivalent sticker*. A multivalent ligand that binds to sticker and spacer sites on scaffolds is designated as *multivalent bipartite*. And finally, a multivalent ligand that binds to spacer sites on scaffolds is designated as *multivalent spacer*. Here, the assumption is the valence of the ligand is always lower than the scaffold sticker valence and the ligand cannot drive phase separation on its own.

In its simplest formalism, polyphasic linkage makes the implicit assumption that ligands do not change the concentrations of scaffolds within dense phases.^20^ Computational studies suggest that this assumption holds for ligands that promote phase separation and the ligand-to-scaffold concentration ratio does not greatly exceed one.^45^ Further, the theory does not provide a set of rules regarding ligand features that lead to the suppression vs promotion of scaffold phase separation. These considerations are important for building on the polyphasic formalism to design ligands that control scaffold phase behavior in a prescribed manner. Numerical simulations help fill this void, and this has been demonstrated by the development and deployment of different flavors of coarse-grained models.^43,45,64,65^ In these models, scaffolds are described using *stickers-*and*-spacers* architectures whereby scaffolds are modeled either as patchy particles [Fig. 4(a)] or linear polymers [Fig. 4(b)].^27,66^ Stickers are sites or motifs that engage in specific inter-scaffold physical crosslinks that are reversible. Spacers are interspersed between stickers, and the solvation preferences of spacers, specifically their excluded volumes (also referred to as effective solvation volumes), determine whether phase separation aids percolation or if phase separation is destabilized such that percolation occurs without phase separation.^27,67^

In general, phase separation driven by homotypic interactions among scaffold molecules can be regulated by the following features of ligands: (1) the relative valence of specific binding sites on ligands vs the sticker valence of scaffolds, (2) whether the ligand sites bind to sticker vs spacer sites on the scaffold, (3) the relative affinities of inter-scaffold interactions vs scaffold-ligand interactions, and (4) the relative concentrations of scaffold to ligands [Fig. 4(c)]. It is worth noting that our findings and the rules we summarize below are strictly true if the sticker valence does not change upon forming a condensate or ligand binding. Accordingly, the rules we summarize are valid in the absence of ligand-induced conformational changes or changes to assembly states that alter the intrinsic valence of stickers.

Working within the caveats stated above, monovalent ligands suppress scaffold phase separation regardless of whether they bind specifically to scaffold stickers or spacers.^45^ For monovalent ligands that bind scaffold stickers, suppression occurs through direct binding and thus competition with scaffold–scaffold interactions. Monovalent ligands that bind scaffold spacers suppress scaffold phase separation by enhancing the excluded volumes of spacers.^27,45^ Multivalent ligands that bind to scaffold stickers, but have a lower valence than the scaffold, generally suppress scaffold phase separation by replacing some of the inter-scaffold crosslinks with ligand–scaffold crosslinks.^45,65^ However, the relative strengths of the scaffold–ligand and scaffold–scaffold interactions also contribute to the impact of multivalent ligands. Specifically, as the scaffold–ligand interaction affinity increases past that of the scaffold–scaffold interaction, the suppressing effect of multivalent ligands that bind stickers is weakened, and these ligands may even promote scaffold phase separation.^45,64^ Multivalent ligands that bind specifically to scaffold spacers promote scaffold phase separation by contributing additional crosslinks among scaffold molecules.^45,65^ However, at high ligand concentrations, even multivalent ligands that bind specifically to scaffold spacers can start to dilute scaffold–scaffold interactions and eventually suppress scaffold phase separation [Fig. 4(c)].^45^

Multivalent ligands that bind to sticker and spacer sites can feature a large range of regulatory effects that are dependent on the relative affinities of the ligand–scaffold sticker, ligand–scaffold spacer, and scaffold–scaffold interactions.^45^ For the case of a divalent ligand where one site binds specifically to scaffold stickers and the other site binds specifically to scaffold spacers, one can realize an array of modulatory behaviors through ligands. If both types of ligand–scaffold interactions are weaker than the scaffold–scaffold interaction, then the ligand does not change the drive of the scaffold to undergo phase separation. In contrast, if the ligand–scaffold interaction involving scaffold stickers is stronger than the other interactions, the ligand suppresses scaffold phase separation. Finally, if the ligand–scaffold interactions are greater than or equal to the scaffold–scaffold interactions or the ligand–scaffold spacer interaction is the strongest, then the ligand tends to promote scaffold phase separation. However, at high ligand concentrations these divalent ligands begin to suppress scaffold phase separation [Fig. 4(c)]. The relative valencies of the number of ligand sites that bind scaffold stickers vs spacers is also a parameter than can be tuned to control scaffold phase behavior for multivalent ligands that bind stickers as well as spacers sites.

**FIG. 5.**
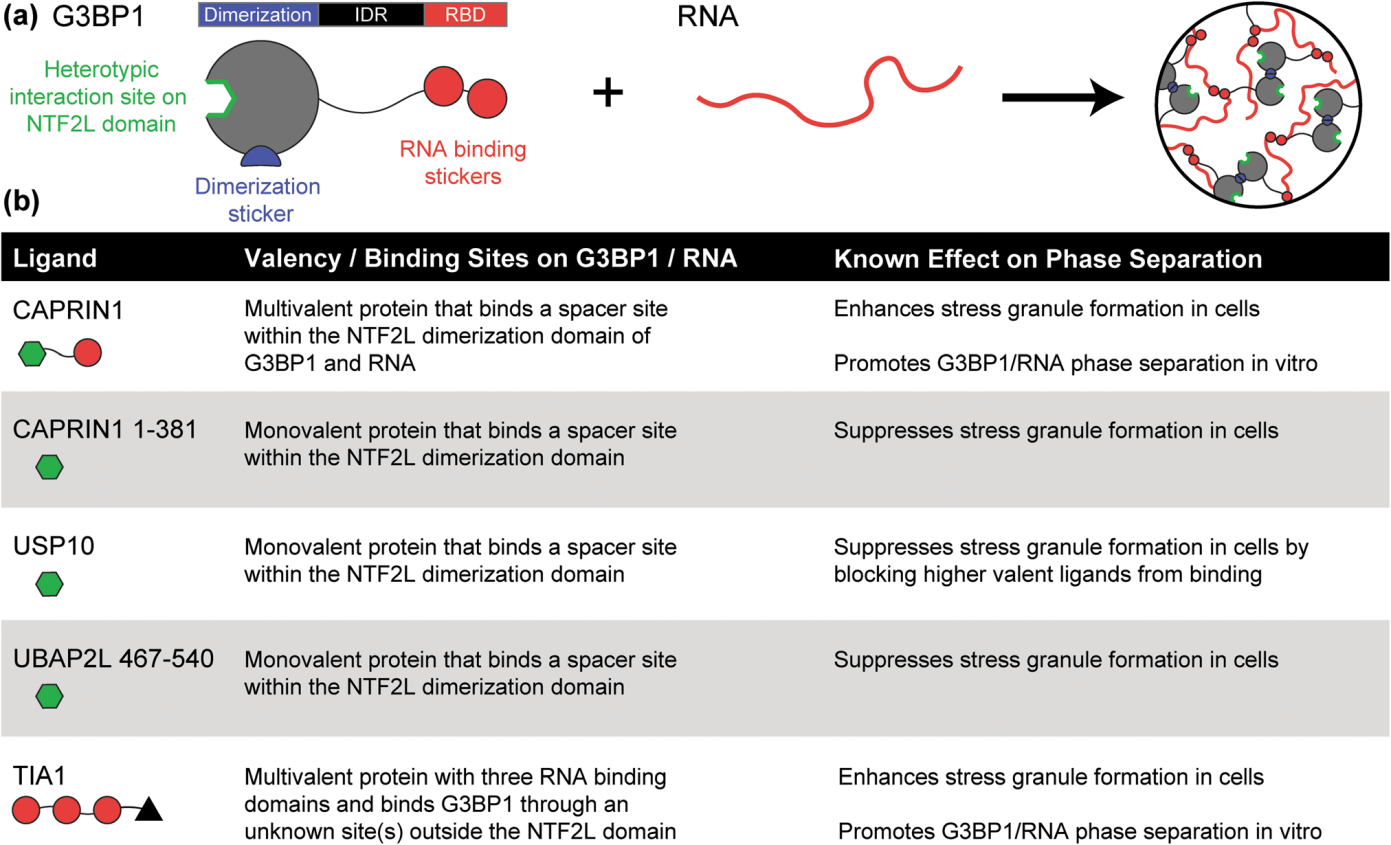
Summary of the effect of ligands on the phase behavior of stress granule scaffolds. (a) Schematic of the scaffolds that drive stress granule formation. G3BP1 interacts with itself through its dimerization domain and interacts with RNA through its RNA binding domain (RBD) to drive phase separation. (b) Features of known ligands and their effects on stress granule formation. The table summarizes the effects that have been documented for five different ligands.^15,17^ The green hexagon implies the ligand has a site that binds to the dimerization domain of G3BP1, albeit to a site that lies outside the region that drives homotypic interactions. Red circles imply that the ligand binds RNA. The black triangle indicates that TIA1 interacts with G3BP1 through an unknown interaction site outside the dimerization domain.

Rules regarding the features of ligands that contribute to suppression vs promotion of scaffold phase separation are concordant with recent studies that have examined the effects of naturally occurring ligands on stress granules.^7,15,17^ These condensates are cytoplasmic bodies that form in response to different types of stress. Their major role appears to be the sequestration of naked unfolded RNA (nuRNA) molecules that are released from the runoff of polysomes.^68^ Further, the organization of proteins within stress granules appears to help in minimizing the entanglements of nuRNA molecules.^7^ Three studies, published simultaneously, have helped clarify the molecular driving forces that underlie stress granule formation.^7,15,17^ The key scaffolds are G3BP1 and its paralog G3BP2—referred to jointly as G3BP1/2—and nuRNA molecules. Phase separation is driven partly by dimerization/oligomerization of G3BP1/2. This increases the valence of the RNA binding domains (RBDs) and the C-terminal RG-rich motifs that help drive phase separation through a network of protein–RNA interactions involving G3BP1/2 and nuRNA molecules. Thus, G3BP1/2 has at least one sticker region for homotypic interactions in its NTF2L dimerization/oligomerization domain and two sticker regions that promote heterotypic interactions with nuRNA molecules [Fig. 5(a)]. The disordered region that connects the dimerization and RNA binding domains is akin to a spacer region that regulates the phase behavior of G3BP1/2-nuRNA mixtures, but it does not harbor sticker sites for either homotypic or heterotypic interactions that contribute to phase separation.

Multivalent ligands that bind at least one spacer site on G3BP1/2 and can bridge G3BP1/2 and RNA by binding RNA through one or more RBDs promote G3BP1/2-RNA phase separation *in vitro* and enhance stress granule formation in cells [Fig. 5(b)].^15,17^ In contrast, monovalent ligands, such as those in which the RBDs are removed, suppress stress granule formation in cells [Fig. 5(b)].^17^ These results suggest that the rules that have been uncovered regarding the features of ligands that suppress or promote phase separation are transferrable to systems where phase separation is driven by a combination of homotypic and heterotypic interactions.

## VI. LIGANDS AS THERAPEUTICS

**FIG. 6.**
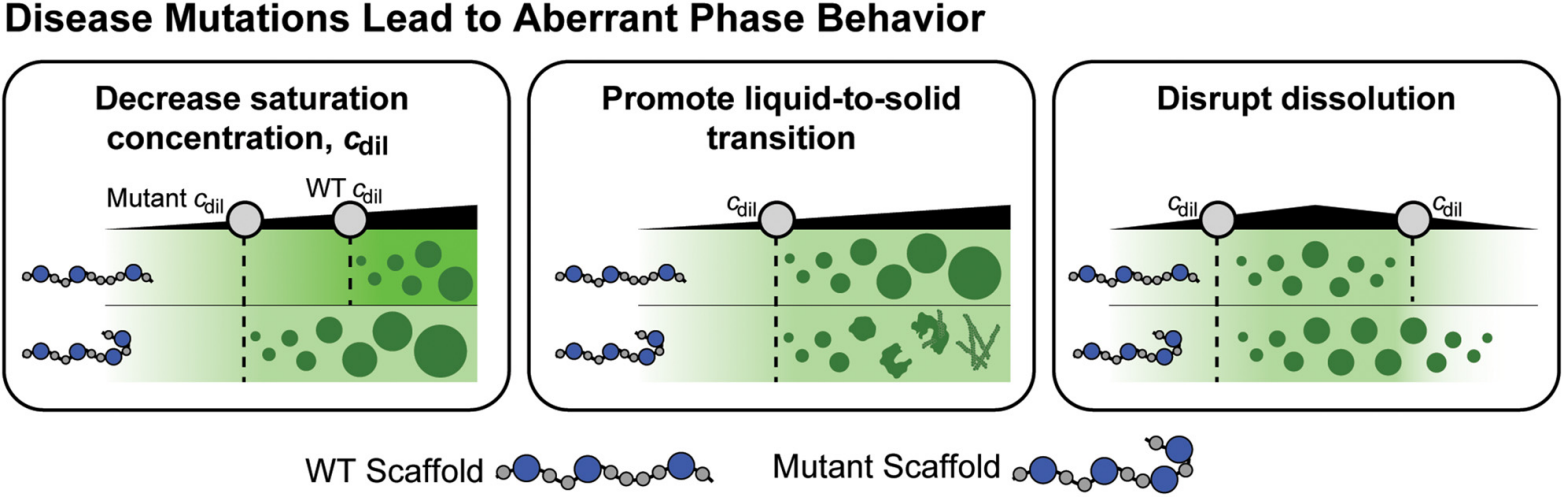
Schematic of how disease-associated mutations change the phase behaviors of scaffolds. Here, we represent a mutation as an insertion of an additional sticker site in the scaffold. Black triangles denote the input scaffold concentration. Disease-associated mutations have been observed to reduce *c*_dil_, promote a liquid-to-solid transition, and disrupt dissolution.

Many disease-associated mutations in scaffold molecules lead to aberrant phase behavior that includes enhanced driving forces for phase separation,^30,49^ dynamical arrest of condensates,^69,70^ and liquid-to-solid transitions with condensates serving as crucibles for these transitions ^49,71–78^ (Fig. 6).

Given the connection between disease-associated mutations and changes in phase behavior, it follows that regulating the formation and/or dissolution of condensates using ligands may be a beneficial therapeutic approach for diseases such as amyotrophic lateral sclerosis (ALS), Huntington's disease, and certain types of cancers.^49,71–78^ In Huntington's disease, the mutant huntingtin protein promotes homotypic phase separation. However, overexpression of the protein profilin reduces mutant huntingtin aggregation and toxicity in cells.^79,80^ *In vitro* experiments have shown that profilin acts by preferentially binding monomers and oligomers to suppress the ability of mutant huntingtin to undergo transitions to spherical and fibrillar phases.^44^

The work of Posey *et al.*^44^ is noteworthy for several reasons: They show how saturation concentrations can be measured using simple, cuvette-based right angle static light scattering measurements. These measurements, performed in the absence of profilin and increasing concentrations of profilin, provide a clear readout of how phase separation is destabilized by preferential binding to the dilute M-phase for the exon 1 encoded fragment of huntingtin (Httex1). Further, Posey *et al.* use a fluorescence-based assay combined with linkage theory and coarse-grained simulations to understand how profilin acts to alter Httex1 phase behavior. The work of Posey *et al.*^44^ lays out the series of experiments combined with analyses that can be brought to bear for uncovering mechanistic details and a comprehensive understanding of how system-specific, ligand-mediated modulation of phase separation can be achieved via polyphasic linkage.

Studies focused on modulating the phase behavior of the protein fused in sarcoma (FUS) have demonstrated the potential benefits of using ligands as a therapeutic strategy for neurodegenerative diseases.^69,81,82^ Mutations in FUS are associated with ALS and frontotemporal dementia.^83^ The addition of Kapβ2 as a ligand dissolves condensates and hydrogels formed via homotypic interactions among FUS molecules.^50^ Wheeler *et al.* showed that lipoamide/lipoic acid can be used to regulate phase behaviors of disease-associated mutants of FUS.^82^ These drugs appear to increase the barrier to aberrant liquid-to-solid transitions that are associated with the G156E mutation. Additionally, lipoamide and lipoic acid also enabled the reversal of motor defects in flies expressing FUS with ALS related mutations.

The design of scaffold-specific ligands might provide a new route to intervene therapeutically by impacting processes such as phase separation. Ligands can be identified through screens that assess the effect of each ligand on scaffold phase separation. Alternatively, as summarized in Sec. V, rules that have been uncovered can be brought to bear in supervised designs of ligand libraries that either suppress or promote scaffold phase behavior.

## VII. NEW DIRECTIONS

### A. Ligand effects on condensates that are driven by heterotypic interactions

Sections II, V, and VI summarized polyphasic linkage concepts and insights regarding the features of ligands that modulate the phase behavior where phase separation involves a single type of scaffold molecule and the driving forces are effectively homotypic interactions. In systems where heterotypic interactions are the main drivers of phase separation, the polyphasic linkage formalism requires generalization, since the contributions to binding polynomials involve multiple species. This can be illustrated using new sets of preliminary simulations where we model phase separation as being the result of purely heterotypic interactions and assess the impact of ligands on this type of phase behavior.

**FIG. 7.**
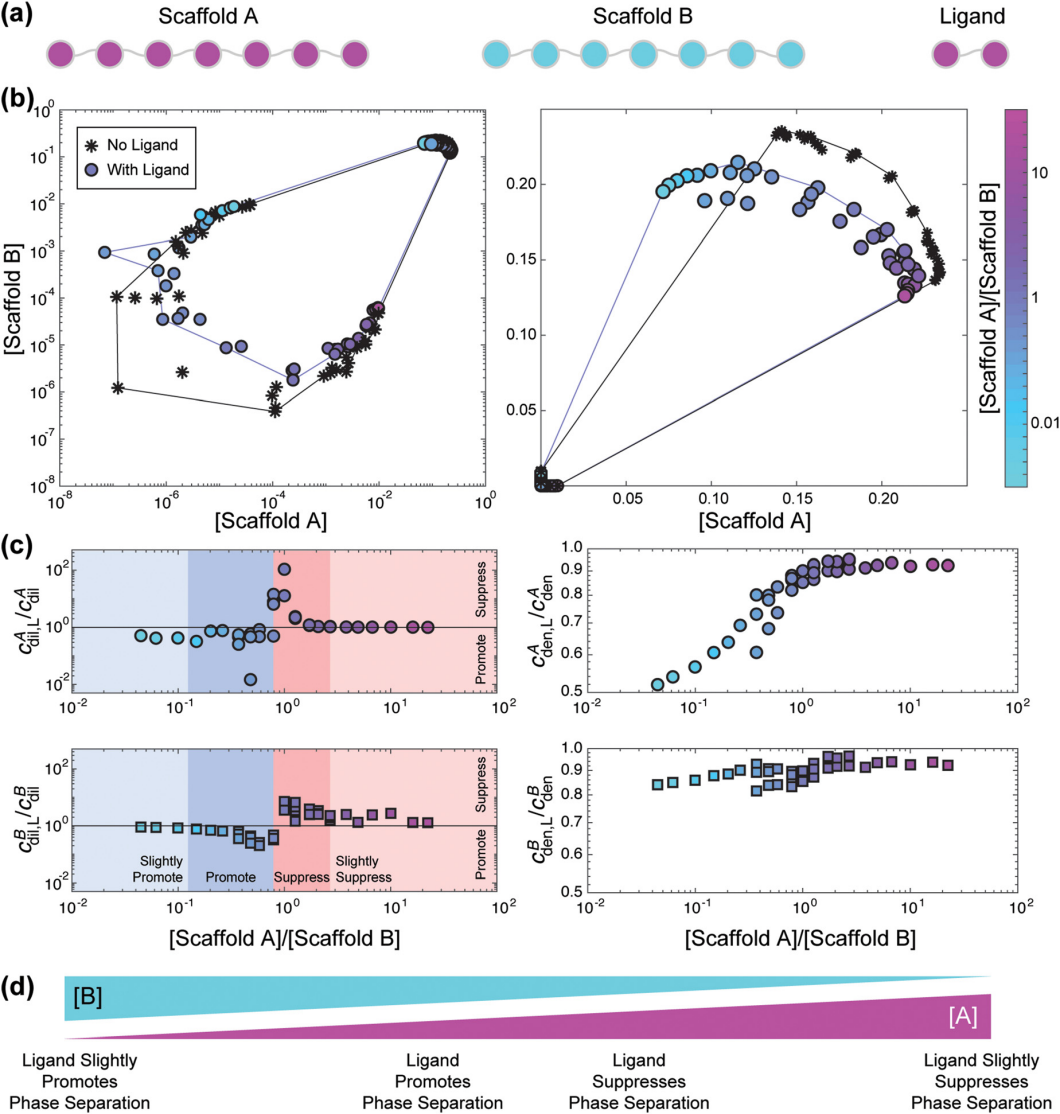
Example effect of a ligand on the phase behavior of a linear polymer model with purely heterotypic interactions. (a) Schematic of model system. Each scaffold has seven sticker sites. The ligand has two sticker sites, which are the same as scaffold A and thus can only bind scaffold B. (b) Phase boundaries of the system without (black, stars) and with (purple, circles) the ligand shown in log and linear scale. Here, concentration is in stickers/lattice sites. Log scale allows for viewing changes in *c*_dil_, whereas linear scale allows for viewing changes in *c*_den_. The color of the circles for the ligand case correspond to the ratio of the input concentrations of the scaffolds, [scaffold A]/[scaffold B] When the input concentration has an excess of scaffold A, the color is pinker, and when the input concentration has an excess of scaffold B, the color is more blue-green. Simulations were performed using the lattice simulation engine LASSI as described in Ruff *et al.*^45^ All sticker–sticker interaction energies were set to −2/T*, where T* is the effective temperature. The ligand concentration was set to 1e-3 molecules/lattice sites. (c) Change in *c*_dil_ and *c*_den_ for each scaffold molecule as a function of the ratio of the input concentrations, [scaffold A]/[scaffold B]. If *c*_dil,L_*/c*_dil_ is less than one, then the ligand promotes phase separation of that scaffold, whereas if *c*_dil,L_*/c*_dil_ is greater than one, then the ligand suppresses phase separation of that scaffold. When taking account the shifts for both scaffold A and B, we find that when [scaffold A]/[scaffold B] is large, the ligand slightly suppresses phase separation (light red shade). This suppression increases as [scaffold A] approaches [scaffold B] (darker red shade). When [scaffold B] is slightly larger than [scaffold A], the ligand has the greatest effect at promoting phase separation (dark blue shade). When [scaffold B] is much larger than [scaffold A], then the ligand slightly promotes phase separation (light blue shade). In regard to the dense phase, the concentration of scaffold B in the dense phase shows limited change in the presence of the ligand, whereas the concentration of scaffold A in the dense phase decreases as [scaffold A]/[scaffold B] decreases. (d) Schematic summarizing the results shown in panels (a)–(c).

Figure 7 shows how the inclusion of a ligand modulates purely heterotypic phase separation as assessed in a lattice simulation.^66^ Each scaffold has seven sticker sites that makes a single reversible cross-link with a site on the other scaffold [Fig. 7(a)]. The phase boundary for this system shows closed loop behavior as expected for purely heterotypic interactions [Fig. 7(b), black]. Here, [scaffold A] and [scaffold B] refer to the total concentrations, in units of stickers per lattice sites, of scaffold molecules A and B, respectively. To assess the effect of a ligand on overall phase behavior, we included a ligand with two binding sites that are identical to the stickers of scaffold A [Fig. 7(a)]. Such a system mimics the scaffold-client systems studied by Banani *et al.*,^13^ although the concentrations of client molecules in their work were low enough to ensure that the client does not behave like a modulatory ligand.

Our simulations show that the addition of a low-valence ligand can suppress or promote scaffold phase separation depending on the relative total concentrations of scaffold A and scaffold B [Fig. 7(b)–7(c)]. In the presence of a large excess of scaffold A, the ligand can suppress phase separation as shown by the observation that $\left(\frac{c_{\text{dil},L}^{B}}{c_{\text{dil}}^{B}}\right)>1$ [Fig. 7(c), light red]. This is because the sub-stoichiometric amount of scaffold B is partially sequestered by interactions with the ligand, thus requiring a higher overall concentration of scaffold B to drive phase separation [Fig. 7(c), squares, light red].

For a two-scaffold system with two scaffolds of equal effective valence that undergoes phase separation purely via obligate heterotypic interactions, such as the system of study here, $c_{\text{dil}}^{A}$ and $c_{\text{dil}}^{B}$ are minimized for a 1:1 stoichiometry of the scaffolds. This arises when (scaffold A) = (scaffold B) [Fig. 7(b), black].^8,27^ Ligands with two binding sites that are identical to the stickers of scaffold A have the largest effect on modulating phase separation when [scaffold A] = [scaffold B] [Fig. 7(c), dark red and dark blue]. Accordingly, the relevant parameter is the input stoichiometric ratio of scaffolds A and B, which we define as $r=\frac{\left[\text{scaffold}\;A\right]}{\left[\text{scaffold}\;B\right]}$. As *r* decreases from ∼2 to ∼0.8, the ability of the ligand to suppress phase separation is maximized as evidenced by the fact that $\left(\frac{c_{\text{dil},L}}{c_{\text{dil}}}\right)>1$ for both scaffolds [Fig. 7(c), dark red]. However, as *r* decreases below 0.8, the presence of the ligand leads to a promotion of scaffold phase separation whereby $\left(\frac{c_{\text{dil},L}}{c_{\text{dil}}}\right)<1$ for both scaffolds A and B [Fig. 7(c) dark blue]. The promotion of phase separation in this regime arises from the fact that the ligand renormalizes the concentration of scaffold A. When there is a large excess of scaffold B, the ligand has a significant modulatory effect on the concentrations of scaffold molecules in the dense phase [Fig. 7(c)] Although the presence of the ligand always decreases $c_{\text{den}}^{A}$ and $c_{\text{dil}}^{B}$, $c_{\text{den}}^{A}$ shows the largest decrease when *r* < 1.

When phase separation is driven by heterotypic rather than homotypic interactions, additional variables influence how a ligand regulates scaffold phase separation. These variables include the relative total concentrations of the scaffold molecules and which scaffold molecule the ligand binds. Low-valence ligands are not general suppressors of phase separation as was observed for the case of purely homotypic interactions. For instance, when the ligand is a lower-valence version of one of the scaffolds, the ligand can also engage in heterotypic interactions that can suppress or support networking. Therefore, the ability to suppress or promote phase separation will depend on the relative input concentrations of the scaffold molecules.

### B. Ligand effects on condensates that are driven by homotypic and heterotypic interactions

Many cellular condensates are driven by a combination of homotypic and heterotypic interactions. For instance, in stress granule formation G3BP1 interacts with itself through its NTF2L dimerization domain and engages in heterotypic interactions with nuRNA molecules. Although, heterotypic interactions with RNA are dominant determinants of phase separation, the dimerization domain of G3BP1 is necessary although insufficient for phase separation.^7,17,40^ Additionally, some of the putative scaffolds of P-bodies engage in homotypic interactions, including Edc3.^14,84–86^ Homotypic and heterotypic interactions of NPM1 may also be important for the formation of the granular component of the nucleolus.^87,88^

**FIG. 8.**
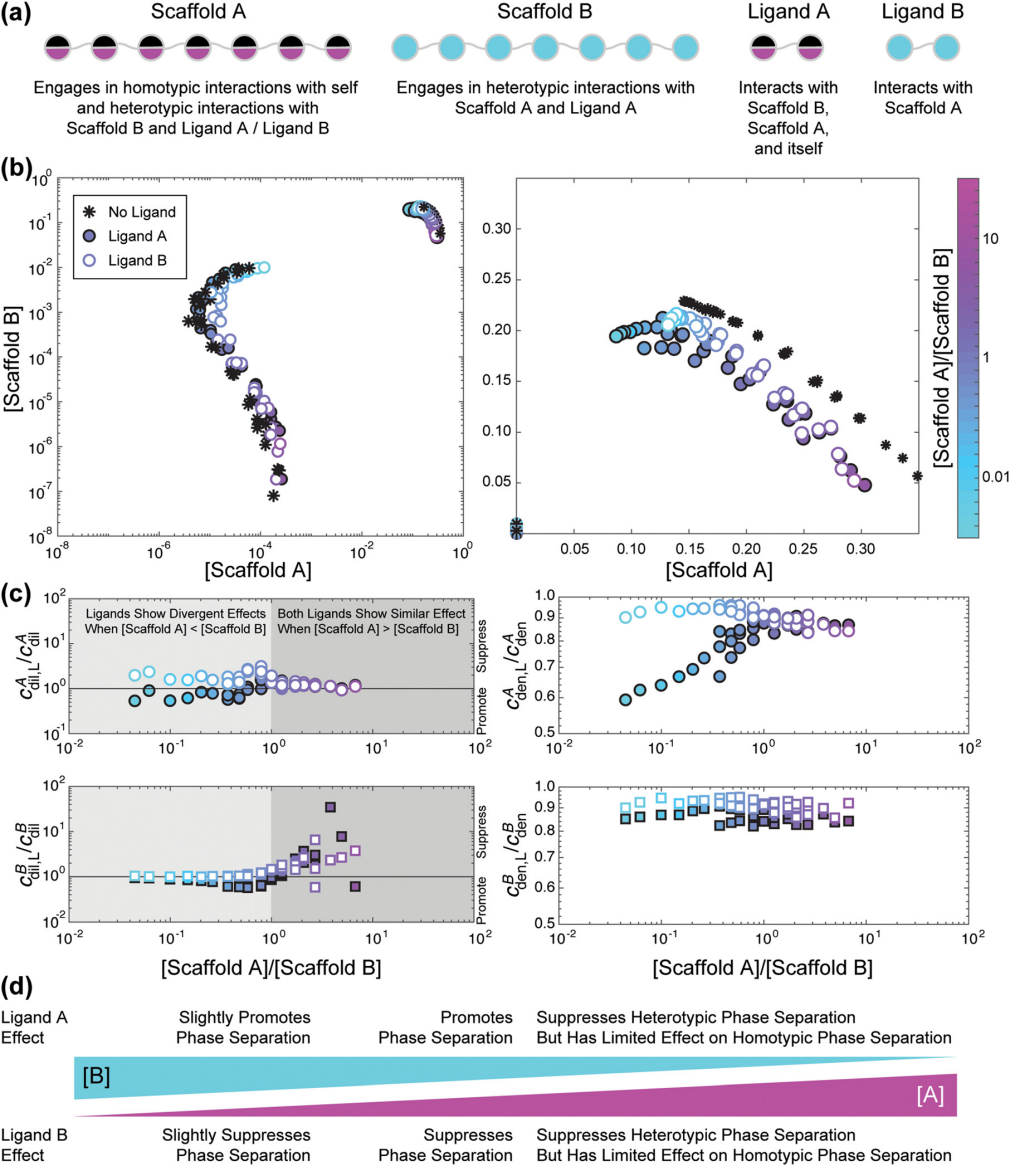
Example effect of ligands on the phase behavior of a linear polymer model with homotypic and heterotypic interactions. (a) Schematic of model system. Each scaffold has seven sticker sites. Scaffold A can interact with itself or with scaffold B, whereas scaffold B can only engage in heterotypic interactions with scaffold A. The two ligands examined were divalent versions of scaffold A and scaffold B. (b) Phase boundaries of the system without (black, stars) and with ligand A (filled-in circles) and with ligand B (empty circles) shown in log and linear scale. Here, concentration is in units of stickers/lattice sites. The log scale allows for viewing changes in *c*_dil_, whereas the linear scale allows for viewing changes in *c*_den_. The color of the circles for simulations in the presence of ligand correspond to the input stoichiometric ratio *r*. When the input concentration involves an excess of scaffold A, the color is pinker, and when the input concentration involves an excess of scaffold B, the color is blue-green. Simulations were performed using the lattice simulation engine LASSI as described in Ruff *et al.*^45^ All sticker–sticker interaction energies were set to −2/T*, where T* is the effective temperature. The ligand concentration was set to 1e-3 molecules/lattice sites. (c) Changes in *c*_dil_ and *c*_den_ for each scaffold molecule as a function of the ratio of *r*. If *c*_dil,L_*/c*_dil_ is less than one, then the ligand promotes phase separation of that scaffold, whereas if *c*_dil,L_*/c*_dil_ is greater than one, then the ligand suppresses phase separation of that scaffold. (d) Summary schematic of the above results.

We next assessed how ligands impact phase behavior when there is an interplay between homotypic and heterotypic interactions. These results are summarized in Fig. 8. Here, scaffold A can also engage in homotypic interactions [Fig. 8(a)] and the interaction strengths of the homotypic and heterotypic interactions are set to be equal. In the absence of a ligand, the phase boundary is no longer a closed loop [Fig. 8(b), black stars]. This is because when scaffold A is in excess, scaffold A can still phase separate through homotypic interactions. We assess the effects of two types of ligands on the joint phase behavior of the two scaffolds. The ligands considered include a divalent ligand with sites that are identical to those of the stickers on scaffold A. This is denoted as ligand A. The ligand designated as B is also divalent, and the binding sites are identical to those of the stickers on scaffold B [Fig. 8(a)]. When *r* > 1 and the total concentration of scaffold A is greater than that of scaffold B, ligands A and B have similar effects on the phase behavior of the scaffolds [Fig. 8(b)–8(c)]. Specifically, $c_{\text{dil}}^{A}$ changes minimally whereas $c_{\text{dil}}^{B}$ increases [Fig. 8(c)]. The implication is that neither of the ligands have a significant influence on the homotypic interactions among scaffold A molecules. In contrast, both ligands reduce the ability of scaffold B to co-phase separate. This is because ligand A interacts preferentially with scaffold B in the dilute phase and sequesters it from the dense phase, whereas ligand B competes with scaffold B for heterotypic interactions with scaffold A in the dense phase, leading to an upshift in $c_{\text{dil}}^{B}$.

The effects of the two ligands diverge when *r* is less than one [Fig. 8(b)–8(c)]. When the total concentration of scaffold B is greater than that of scaffold A, ligand A promotes scaffold phase separation, whereas ligand B suppresses phase separation [Fig. 8(c)]. The presence of ligand A reduces $c_{\text{dil}}^{A}$ and $c_{\text{dil}}^{B}$, thereby promoting phase separation of both scaffolds. In contrast, the presence of ligand B leads to an increase in $c_{\text{dil}}^{A}$, implying that ligand B sequesters scaffold A in the dilute phase. As the total concentration of scaffold B increases with respect to that of scaffold A, the degrees to which ligands A and B respectively promote vs suppress phase separation decrease [Fig. 8(c)]. Additionally, we find that both ligand types reduce the concentration of scaffolds in the dense phase [Fig. 8(b)–8(c)]. Ligand A always decreases $c_{\text{den}}^{B}$ to a greater extent than ligand B. The largest decrease in scaffold concentration in the dense phase is observed for scaffold A in the presence of ligand A; see results for $c_{\text{den}}^{A}$ in the presence of ligand A, when *r* < 1, i.e., [scaffold A] < [scaffold B]. Given the excess of scaffold B, phase separation is dominated by heterotypic interactions, and this decrease in $c_{\text{den}}^{A}$ mimics the observations for the purely heterotypic case shown in Fig. 7.

Taken together, we find that the precise nature of regulation of collective phase separation in systems with two macromolecular scaffolds will depend on whether phase separation is driven purely by heterotypic interactions or if there is an interplay between homotypic and heterotypic interactions. As a rule, ligands that can contribute additional networking interactions will promote phase separation, whereas ligands that disrupt scaffold networking interactions and cannot compensate for the loss of crosslinks among scaffolds will suppress phase separation. For systems in which phase separation depends, at least partially, on heterotypic interactions, the relative concentrations of the scaffolds and the specificity of ligand sites for scaffold stickers will influence how the ligands affect phase separation. The key finding is that the relative total concentrations of scaffolds will determine how different ligands regulate overall phase behavior.

### C. Networks of ligands

The compositions of biomolecular condensates are heterogeneous and distinctive. Of the tens to hundreds of distinct types of molecules that make up condensates, only a few are likely to be scaffolds that are required for driving phase separation.^15^ Instead, an overwhelming number of macromolecular components of condensates have the potential for being ligands that, depending on their valence, affinities, and concentrations, regulate condensate formation and dissolution. Accordingly, phase separation of condensate driving scaffolds is likely to be influenced by networks of interactions with distinct types of ligands. If condensates consist of ligands that suppress and promote scaffold phase behavior, then there will be an interplay between suppressor vs promoter ligands. Accordingly, cellular control over the expression levels of the two categories of ligands can provide multiple routes to modulating phase behaviors of scaffolds, thereby influencing condensate formation vs dissolution. The ability of ligands to influence the extent of cross-linking among scaffolds will also influence material properties of condensates.

Our understanding of the effects of networks of ligands is being aided by investigations of condensates in live cells, as evidenced by recent efforts directed toward the characterization of stress granules.^15,17^ Yang *et al.*^15^ identified a core stress granule protein–protein interaction network of 36 proteins. Of these, only G3BP1 and G3BP2 were found to be essential for stress granule formation from knockout studies. Of the remaining 34 proteins, several have been found to regulate condensate formation by promoting (CAPRIN-1, UBAP2L, TIA1) or suppressing (USP10) stress granule formation.^15,17,89–92^ Studies suggest that UBAP2L may be a stronger endogenous regulator of stress granule formation than either CAPRIN-1 or USP10.^17,93,94^ These results suggest that cells use the combined contributions of suppressor and promoter ligands to regulate condensate formation and dissolution by controlling the expression levels of different types of ligands.

Multivalent ligands can be designed to either suppress or promote scaffold phase behavior.^45^ Accordingly, multivalent ligands can be designed to program specific responses of condensates. The impacts of designed ligands will be influenced by competing effects of the endogenous ligands. For instance, if a condensate has only one ligand that promotes scaffold phase separation, then a ligand intended to suppress phase separation would need to outcompete only one type of endogenous ligand. Conversely, if a condensate has many endogenous ligands that promote scaffold phase separation, then the designed ligand must outcompete the collective effects of the endogenous ligands. It is also important to note that proper cellular condensate function is likely to depend on more than just the concentration of the scaffold(s) in the dilute and dense phases. For instance, condensate function may depend on maintaining the endogenous composition as well as relative and/or total concentrations of non-scaffold molecules within the condensate.^95,96^ Thus, it is important to make sure that designed ligands do not disrupt functionally relevant compositional profiles of condensates.

### D. Annotating condensate proteomes to identify ligands and predict their effect on scaffold phase behavior

Ongoing efforts are focused on identifying macromolecular components of biomolecular condensates and quantifying their relative abundance.^15,41,42,90,92,97^ Particular attention is being paid to identifying the macromolecular compositions of stress granules and P-bodies, and a result of one of these efforts is a curated and scored list found at http://rnagranuledb.lunenfeld.ca/.^92^ Although these lists contain hundreds of distinct molecules, the working hypothesis is that only a subset of these molecules makeup the core stress granule or P-body network.^14,15^

The direct relevance of polyphasic linkage concepts raises the following question: Given knowledge of the composition profiles of condensates, how would we classify macromolecular components of condensates as scaffolds, suppressor ligands, driver ligands, and passive clients? The approach of Sanders *et al.*^17^ points to a useful route: they used a combination of deletion constructs and sequence analysis to identify non-scaffold molecules within stress granules that interact with the dimerization domain of G3BP1 and either suppress or promote stress granule formation.^17^ They predicted and showed that proteins that bound G3BP1 and had an RNA binding domain would promote stress granule formation, whereas proteins that bound G3BP1 but lacked an RNA binding domain would act to suppress stress granule formation. These results suggest that if one has information about how the scaffolds drive phase separation, then one can use sequence features of the interaction networks within condensates to identify putative ligands based on the numbers and types of scaffold binding sites. These classifiers should pave the way for predicting and modeling how a putative ligand and its expression level contributes to condensate formation or dissolution.

Reconstitution and examination of the phase behaviors of multicomponent systems should help in defining the interplay between scaffolds and putative ligands.^84^ These types of studies are likely to be beneficial for condensates in which a well-defined scaffold set has not been identified. For instance, Xing *et al.* identified seven P-body proteins that were highly concentrated within the condensate.^14^ Of these proteins, five had been previously identified as putative scaffolds based on the observation that deletion of these proteins disrupted P-body formation. However, given the degree of disruption, results suggest that many of these molecules may fall within the spectrum of ligand to scaffold. Xing *et al.* suggested that deletion of proteins that are more scaffold-like should change *c*_dil_ and *c*_den_ for a large set of the other condensate components, whereas deletion of proteins that are more ligand-like should have a weaker and more targeted effect. These hypotheses can be tested using a combination of numerical simulations, generalizations of polyphasic linkage theory, and targeted experiments.

## VIII. CONCLUSIONS

We have highlighted the importance and relevance of the concepts of polyphasic linkage for describing how ligands afford control over biomolecular condensates through control over scaffold phase separation. The recent adaptation of this established concept has yielded important insights regarding the features of ligands that are relevant for their behavior as suppressors vs promoters of phase separation. However, advances are needed to understand ligand effects on more complicated, biologically relevant systems. These systems include scaffolds with different types of stickers that have distinct ligand binding affinities, networks of ligands, and multiple scaffolds defined by combinations of homotypic and heterotypic interactions.

Efforts that combine experiments, theory, and simulations will help obtain a holistic understanding of how a ligand impacts phase separation of a multivalent macromolecular scaffold. We need to be able to measure scaffold concentrations in dilute and dense phases, to measure affinities of ligand binding in dense and dilute phases, and to fit scaffold concentration data to linkage theory. Furthermore, the use of simulations is likely to provide a coherent interpretation of the totality of the data. Posey *et al.* have demonstrated how a suite of experiments, theory-driven analysis, and simulations can be brought to bear. They focused on a system where Httex1 was the multivalent macromolecular scaffold and profilin was the ligand. Four decades after it was formulated, the application of polyphasic linkage theory is in still in its infancy. The work of Posey *et al.*^44^ provides a template for how this framework can be applied to a range of other systems, while also paving the way for advances that take on the challenge of understanding more complicated biologically relevant systems.

## AUTHORS' CONTRIBUTIONS

All three authors have been actively involved in adapting polyphasic linkage concepts for describing ligand-mediated effects on phase separation of multivalent macromolecules. K.M.R. and R.V.P. did most of the writing and integration of this review with extant literature. F.D. performed and analyzed LASSI-based simulations. K.M.R. prepared all the figures. R.V.P. secured funding. All authors read and contributed edits to the final version of the manuscript.

## Data Availability

The data that support the findings in this paper are available upon reasonable request from the corresponding author.

## References

[1] S. F. Banani , H. O. Lee , A. A. Hyman , and M. K. Rosen , Nat. Rev. Mol. Cell. Biol. 18(5), 285–298 (2017). 10.1038/nrm.2017.7 28225081 PMC7434221

[2] J. B. Woodruff , A. A. Hyman , and E. Boke , Trends Biochem. Sci. 43(2), 81–94 (2018). 10.1016/j.tibs.2017.11.005 29258725

[3] H. Zhang , S. Elbaum-Garfinkle , E. M. Langdon , N. Taylor , P. Occhipinti , A. A. Bridges , C. P. Brangwynne , and A. S. Gladfelter , Mol. Cell. 60(2), 220–230 (2015). 10.1016/j.molcel.2015.09.017 26474065 PMC5221516

[4] C. P. Brangwynne , C. R. Eckmann , D. S. Courson , A. Rybarska , C. Hoege , J. Gharakhani , F. Julicher , and A. A. Hyman , Science 324(5935), 1729–1732 (2009). 10.1126/science.1172046 19460965

[5] X. Su , J. A. Ditlev , E. Hui , W. Xing , S. Banjade , J. Okrut , D. S. King , J. Taunton , M. K. Rosen , and R. D. Vale , Science 352(6285), 595–599 (2016). 10.1126/science.aad9964 27056844 PMC4892427

[6] P. Anderson and N. Kedersha , Cell Stress Chaperones 7(2), 213–221 (2002). 12380690 10.1379/1466-1268(2002)007<0213:vstroe>2.0.co;2 PMC514820

[7] J. Guillen-Boixet , A. Kopach , A. S. Holehouse , S. Wittmann , M. Jahnel , R. Schlussler , K. Kim , I. Trussina , J. Wang , D. Mateju , I. Poser , S. Maharana , M. Ruer-Gruss , D. Richter , X. Zhang , Y. T. Chang , J. Guck , A. Honigmann , J. Mahamid , A. A. Hyman , R. V. Pappu , S. Alberti , and T. M. Franzmann , Cell 181(2), 346–361.E17 (2020). 10.1016/j.cell.2020.03.049 32302572 PMC7181197

[8] P. Li , S. Banjade , H. C. Cheng , S. Kim , B. Chen , L. Guo , M. Llaguno , J. V. Hollingsworth , D. S. King , S. F. Banani , P. S. Russo , Q. X. Jiang , B. T. Nixon , and M. K. Rosen , Nature 483(7389), 336–340 (2012). 10.1038/nature10879 22398450 PMC3343696

[9] I. A. Sawyer , G. L. Hager , and M. Dundr , RNA Biol. 14(6), 791–803 (2017). 10.1080/15476286.2016.1243648 27715441 PMC5519236

[10] J. Berry , S. C. Weber , N. Vaidya , M. Haataja , and C. P. Brangwynne , Proc. Natl. Acad. Sci. U. S. A. 112(38), E5237–E5245 (2015). 10.1073/pnas.1509317112 26351690 PMC4586886

[11] S. P. Shevtsov and M. Dundr , Nat. Cell Biol. 13(2), 167–173 (2011). 10.1038/ncb2157 21240286

[12] A. K. Rai , J. X. Chen , M. Selbach , and L. Pelkmans , Nature 559(7713), 211–216 (2018). 10.1038/s41586-018-0279-8 29973724

[13] S. F. Banani , A. M. Rice , W. B. Peeples , Y. Lin , S. Jain , R. Parker , and M. K. Rosen , Cell 166(3), 651–663 (2016). 10.1016/j.cell.2016.06.010 27374333 PMC4967043

[14] W. Xing , D. Muhlrad , R. Parker , and M. K. Rosen , Elife 9, e56525 (2020). 10.7554/eLife.56525 32553117 PMC7373430

[15] P. Yang , C. Mathieu , R. M. Kolaitis , P. Zhang , J. Messing , U. Yurtsever , Z. Yang , J. Wu , Y. Li , Q. Pan , J. Yu , E. W. Martin , T. Mittag , H. J. Kim , and J. P. Taylor , Cell 181(2), 325–345 (2020). 10.1016/j.cell.2020.03.046 32302571 PMC7448383

[16] M. Feric , N. Vaidya , T. S. Harmon , D. M. Mitrea , L. Zhu , T. M. Richardson , R. W. Kriwacki , R. V. Pappu , and C. P. Brangwynne , Cell 165(7), 1686–1697 (2016). 10.1016/j.cell.2016.04.047 27212236 PMC5127388

[17] D. W. Sanders , N. Kedersha , D. S. W. Lee , A. R. Strom , V. Drake , J. A. Riback , D. Bracha , J. M. Eeftens , A. Iwanicki , A. Wang , M. T. Wei , G. Whitney , S. M. Lyons , P. Anderson , W. M. Jacobs , P. Ivanov , and C. P. Brangwynne , Cell 181(2), 306–324 (2020). 10.1016/j.cell.2020.03.050 32302570 PMC7816278

[18] A. G. Larson , D. Elnatan , M. M. Keenen , M. J. Trnka , J. B. Johnston , A. L. Burlingame , D. A. Agard , S. Redding , and G. J. Narlikar , Nature 547(7662), 236–240 (2017). 10.1038/nature22822 28636604 PMC5606208

[19] İ. A. Ilik , M. Malszycki , A. K. Lübke , C. Schade , D. Meierhofer , and T. Aktaş , eLife 9, e60579 (2020). 10.7554/eLife.60579 33095160 PMC7671692

[20] J. Wyman and S. J. Gill , Proc. Natl. Acad. Sci. U. S. A. 77(9), 5239–5242 (1980). 10.1073/pnas.77.9.5239 6933555 PMC350033

[21] J. Rupley , J. Mol. Biol. 35(3), 455–476 (1968). 10.1016/S0022-2836(68)80006-3 5673692

[22] M. Rubinstein and A. Dobrynin , Trends Polym. Sci. 5, 181–186 (1997).

[23] A. N. Semenov and M. Rubinstein , Macromolecules 31(4), 1373–1385 (1998). 10.1021/ma970616h

[24] D. Prusty , V. Pryamitsyn , and M. Olvera de la Cruz , Macromolecules 51(15), 5918–5932 (2018). 10.1021/acs.macromol.8b00661

[25] M. Cates and T. Witten , Macromolecules 19(3), 732–739 (1986). 10.1021/ma00157a042

[26] R. D. Groot and W. G. Agterof , J. Chem. Phys. 100(2), 1649–1656 (1994). 10.1063/1.466592

[27] T. S. Harmon , A. S. Holehouse , M. K. Rosen , and R. V. Pappu , Elife 6, e30294 (2017). 10.7554/eLife.30294 29091028 PMC5703641

[28] J. Wang , J. M. Choi , A. S. Holehouse , H. O. Lee , X. Zhang , M. Jahnel , S. Maharana , R. Lemaitre , A. Pozniakovsky , D. Drechsel , I. Poser , R. V. Pappu , S. Alberti , and A. A. Hyman , Cell 174(3), 688–699 (2018). 10.1016/j.cell.2018.06.006 29961577 PMC6063760

[29] J. M. Choi , A. S. Holehouse , and R. V. Pappu , Annu. Rev. Biophys. 49, 107–133 (2020). 10.1146/annurev-biophys-121219-081629 32004090 PMC10715172

[30] Y. Yang , H. B. Jones , T. P. Dao , and C. A. Castañeda , J. Phys. Chem. B 123(17), 3618–3629 (2019). 10.1021/acs.jpcb.9b01024 30925840

[31] E. W. Martin , A. S. Holehouse , I. Peran , M. Farag , J. J. Incicco , A. Bremer , C. R. Grace , A. Soranno , R. V. Pappu , and T. Mittag , Sci. 367(6478), 694–699 (2020). 10.1126/science.aaw8653 PMC7297187 32029630

[32] C. P. Brangwynne , P. Tompa , and R. V. Pappu , Nat. Phys. 11(11), 899–904 (2015). 10.1038/nphys3532

[33] P. J. Flory , J. Chem. Phys. 10(1), 51–61 (1942). 10.1063/1.1723621

[34] I. Alshareedah , M. Muhammad Moosa , and P. R. Banerjee , “Programmable viscoelasticity in protein-RNA condensates with disordered sticker-spacer polypeptides,” bioRxiv. 10.1101/2021.01.24.427968 PMC8595643 34785657

[35] T. M. Franzmann , M. Jahnel , A. Pozniakovsky , J. Mahamid , A. S. Holehouse , E. Nüske , D. Richter , W. Baumeister , S. W. Grill , R. V. Pappu , A. A. Hyman , and S. Alberti , Science 359(6371), eaao5654 (2018). 10.1126/science.aao5654 29301985

[36] H. Cinar , Z. Fetahaj , S. Cinar , R. M. Vernon , H. S. Chan , and R. H. A. Winter , Chem. – A Eur. J. 25(57), 13049–13069 (2019). 10.1002/chem.201902210 31237369

[37] F. G. Quiroz and A. Chilkoti , Nat. Mater. 14(11), 1164–1171 (2015). 10.1038/nmat4418 26390327 PMC4618764

[38] T. J. Nott , E. Petsalaki , P. Farber , D. Jervis , E. Fussner , A. Plochowietz , T. D. Craggs , D. P. Bazett-Jones , T. Pawson , J. D. Forman-Kay , and A. J. Baldwin , Mol. Cell 57(5), 936–947 (2015). 10.1016/j.molcel.2015.01.013 25747659 PMC4352761

[39] S. Elbaum-Garfinkle , Y. Kim , K. Szczepaniak , C. C.-H. Chen , C. R. Eckmann , S. Myong , and C. P. Brangwynne , Proc. Nat. Acad. Sci. USA 112(23), 7189 (2015). 10.1073/pnas.1504822112 26015579 PMC4466716

[40] J. A. Riback , L. Zhu , M. C. Ferrolino , M. Tolbert , D. M. Mitrea , D. W. Sanders , M. T. Wei , R. W. Kriwacki , and C. P. Brangwynne , Nature 581(7807), 209–214 (2020). 10.1038/s41586-020-2256-2 32405004 PMC7733533

[41] S. Jain , J. R. Wheeler , R. W. Walters , A. Agrawal , A. Barsic , and R. Parker , Cell 164(3), 487–498 (2016). 10.1016/j.cell.2015.12.038 26777405 PMC4733397

[42] A. Hubstenberger , M. Courel , M. Bénard , S. Souquere , M. Ernoult-Lange , R. Chouaib , Z. Yi , J. B. Morlot , A. Munier , M. Fradet , M. Daunesse , E. Bertrand , G. Pierron , J. Mozziconacci , M. Kress , and D. Weil , Mol. Cell 68(1), 144–157 (2017). 10.1016/j.molcel.2017.09.003 28965817

[43] A. Ghosh , K. Mazarakos , and H. X. Zhou , Proc. Natl. Acad. Sci. U. S. A. 116(39), 19474–19483 (2019). 10.1073/pnas.1907849116 31506351 PMC6765290

[44] A. E. Posey , K. M. Ruff , T. S. Harmon , S. L. Crick , A. Li , M. I. Diamond , and R. V. Pappu , J. Biol. Chem. 293(10), 3734–3746 (2018). 10.1074/jbc.RA117.000357 29358329 PMC5846159

[45] K. M. Ruff , F. Dar , and R. V. Pappu , Proc. Natl. Acad. Sci. U. S. A. 118(10), e2017184118 (2021). 10.1073/pnas.2017184118 33653957 PMC7958451

[46] A. A. Hyman , C. A. Weber , and F. Jülicher , Annu. Rev. Cell Develop. Biol. 30(1), 39–58 (2014). 10.1146/annurev-cellbio-100913-013325 25288112

[47] W. Tisel , R. Haire , J. White , A. Rosenberg , and C. Middaugh , J. Biol. Chem. 255(19), 8975–8978 (1980). 10.1016/S0021-9258(19)70506-7 7410402

[48] S. Saha , C. A. Weber , M. Nousch , O. Adame-Arana , C. Hoege , M. Y. Hein , E. Osborne-Nishimura , J. Mahamid , M. Jahnel , L. Jawerth , A. Pozniakovski , C. R. Eckmann , F. Jülicher , and A. A. Hyman , Cell 166(6), 1572–1584 (2016). 10.1016/j.cell.2016.08.006 27594427 PMC5034880

[49] T. P. Dao , B. Martyniak , A. J. Canning , Y. Lei , E. G. Colicino , M. S. Cosgrove , H. Hehnly , and C. A. Castaneda , Struct. 27(6), 937–951.E5 (2019). 10.1016/j.str.2019.03.012 PMC6551275 30982635

[50] L. Guo , H. J. Kim , H. Wang , J. Monaghan , F. Freyermuth , J. C. Sung , K. O'Donovan , C. M. Fare , Z. Diaz , N. Singh , Z. C. Zhang , M. Coughlin , E. A. Sweeny , M. E. DeSantis , M. E. Jackrel , C. B. Rodell , J. A. Burdick , O. D. King , A. D. Gitler , C. Lagier-Tourenne , U. B. Pandey , Y. M. Chook , J. P. Taylor , and J. Shorter , Cell 173(3), 677–692 (2018). 10.1016/j.cell.2018.03.002 29677512 PMC5911940

[51] S. Alberti , A. Gladfelter , and T. Mittag , Cell 176(3), 419–434 (2019). 10.1016/j.cell.2018.12.035 30682370 PMC6445271

[52] I. Peran , E. W. Martin , and T. Mittag , Methods Mol. Biol. 2141, 715–730 (2020). 10.1007/978-1-0716-0524-0_37 32696386

[53] N. M. Milkovic and T. Mittag , Methods Mol. Biol. 2141, 685–702 (2020). 10.1007/978-1-0716-0524-0_35 32696384

[54] Y. Lin , D. S. Protter , M. K. Rosen , and R. Parker , Mol. Cell 60(2), 208–219 (2015). 10.1016/j.molcel.2015.08.018 26412307 PMC4609299

[55] B. S. Schuster , E. H. Reed , R. Parthasarathy , C. N. Jahnke , R. M. Caldwell , J. G. Bermudez , H. Ramage , M. C. Good , and D. A. Hammer , Nat. Commun. 9(1), 2985 (2018). 10.1038/s41467-018-05403-1 30061688 PMC6065366

[56] D. Bracha , M. T. Walls , M. T. Wei , L. Zhu , M. Kurian , J. L. Avalos , J. E. Toettcher , and C. P. Brangwynne , Cell 175(6), 1467–1480 (2018). 10.1016/j.cell.2018.10.048 30500534 PMC6724719

[57] Y. Shin , J. Berry , N. Pannucci , M. P. Haataja , J. E. Toettcher , and C. P. Brangwynne , Cell 168(1–2), 159–171 (2017). 10.1016/j.cell.2016.11.054 28041848 PMC5562165

[58] E. Dine , A. A. Gil , G. Uribe , C. P. Brangwynne , and J. E. Toettcher , Cell Syst. 6(6), 655–663 (2018). 10.1016/j.cels.2018.05.002 29859829 PMC6023754

[59] A. Bremer , T. Mittag , and M. Heymann , Lab Chip 20(22), 4225–4234 (2020). 10.1039/D0LC00613K 33057557 PMC7658026

[60] M. R. Kopp , M. Linsenmeier , B. Hettich , S. Prantl , S. Stavrakis , J.-C. Leroux , and P. Arosio , Anal. Chem. 92(8), 5803–5812 (2020). 10.1021/acs.analchem.9b05329 32249573

[61] W. E. Arter , R. Qi , G. Krainer , T. J. Welsh , Y. Xu , P. S. George-Hyslop , S. Alberti , and T. P. J. Knowles , “Rapid generation of protein condensate phase diagrams using combinatorial droplet microfluidics,” bioRxiv. 10.1101/2020.06.04.132308

[62] M. Linsenmeier , M. R. G. Kopp , S. Stavrakis , A. de Mello , and P. Arosio , Biochim. Biophys. Acta. Mol. Cell Res. 1868(1), 118823 (2021). 10.1016/j.bbamcr.2020.118823 32800925

[63] J. B. Woodruff , B. Ferreira Gomes , P. O. Widlund , J. Mahamid , A. Honigmann , and A. A. Hyman , Cell 169(6), 1066–1077 (2017). 10.1016/j.cell.2017.05.028 28575670

[64] V. Nguemaha and H. X. Zhou , Sci. Rep. 8(1), 6728 (2018). 10.1038/s41598-018-25132-1 29712961 PMC5928213

[65] J. R. Espinosa , J. A. Joseph , I. Sanchez-Burgos , A. Garaizar , D. Frenkel , and R. Collepardo-Guevara , Proc. Natl. Acad. Sci. U. S. A. 117(24), 13238–13247 (2020). 10.1073/pnas.1917569117 32482873 PMC7306995

[66] J. M. Choi , F. Dar , and R. V. Pappu , PLoS Comput. Biol. 15(10), e1007028 (2019). 10.1371/journal.pcbi.1007028 31634364 PMC6822780

[67] T. S. Harmon , A. S. Holehouse , and R. V. Pappu , New J. Phys. 20(4), 045002 (2018). 10.1088/1367-2630/aab8d9

[68] N. Kedersha and P. Anderson , in *Methods in Enzymology* ( Academic Press, 2007), Vol. 431, pp. 61–81. 17923231 10.1016/S0076-6879(07)31005-7

[69] A. G. Niaki , J. Sarkar , X. Cai , K. Rhine , V. Vidaurre , B. Guy , M. Hurst , J. C. Lee , H. R. Koh , L. Guo , C. M. Fare , J. Shorter , and S. Myong , Mol. Cell. 77(1), 82–94 (2020). 10.1016/j.molcel.2019.09.022 31630970 PMC6943187

[70] K. Rhine , M. A. Makurath , J. Liu , S. Skanchy , C. Lopez , K. F. Catalan , Y. Ma , C. M. Fare , J. Shorter , T. Ha , Y. R. Chemla , and S. Myong , Mol. Cell 80(6), 1139 (2020). 10.1016/j.molcel.2020.11.031 33338404 PMC7788473

[71] A. Patel , H. O. Lee , L. Jawerth , S. Maharana , M. Jahnel , M. Y. Hein , S. Stoynov , J. Mahamid , S. Saha , T. M. Franzmann , A. Pozniakovski , I. Poser , N. Maghelli , L. A. Royer , M. Weigert , E. W. Myers , S. Grill , D. Drechsel , A. A. Hyman , and S. Alberti , Cell 162(5), 1066–1077 (2015). 10.1016/j.cell.2015.07.047 26317470

[72] T. Murakami , S. Qamar , J. Q. Lin , G. S. Schierle , E. Rees , A. Miyashita , A. R. Costa , R. B. Dodd , F. T. Chan , C. H. Michel , D. Kronenberg-Versteeg , Y. Li , S. P. Yang , Y. Wakutani , W. Meadows , R. R. Ferry , L. Dong , G. G. Tartaglia , G. Favrin , W. L. Lin , D. W. Dickson , M. Zhen , D. Ron , G. Schmitt-Ulms , P. E. Fraser , N. A. Shneider , C. Holt , M. Vendruscolo , C. F. Kaminski , and P. St George-Hyslop , Neuron 88(4), 678–690 (2015). 10.1016/j.neuron.2015.10.030 26526393 PMC4660210

[73] A. Molliex , J. Temirov , J. Lee , M. Coughlin , A. P. Kanagaraj , H. J. Kim , T. Mittag , and J. P. Taylor , Cell 163(1), 123–133 (2015). 10.1016/j.cell.2015.09.015 26406374 PMC5149108

[74] S. L. Crick , K. M. Ruff , K. Garai , C. Frieden , and R. V. Pappu , Proc. Natl. Acad. Sci. U. S. A. 110(50), 20075–20080 (2013). 10.1073/pnas.1320626110 24282292 PMC3864320

[75] T. R. Peskett , F. Rau , J. O'Driscoll , R. Patani , A. R. Lowe , and H. R. Saibil , Mol. Cell 70(4), 588–601 (2018). 10.1016/j.molcel.2018.04.007 29754822 PMC5971205

[76] J. J. Bouchard , J. H. Otero , D. C. Scott , E. Szulc , E. W. Martin , N. Sabri , D. Granata , M. R. Marzahn , K. Lindorff-Larsen , X. Salvatella , B. A. Schulman , and T. Mittag , Mol. Cell 72(1), 19–36 (2018). 10.1016/j.molcel.2018.08.027 30244836 PMC6179159

[77] S. Wegmann , B. Eftekharzadeh , K. Tepper , K. M. Zoltowska , R. E. Bennett , S. Dujardin , P. R. Laskowski , D. MacKenzie , T. Kamath , C. Commins , C. Vanderburg , A. D. Roe , Z. Fan , A. M. Molliex , A. Hernandez-Vega , D. Muller , A. A. Hyman , E. Mandelkow , J. P. Taylor , and B. T. Hyman , EMBO J. 37(7), e98049 (2018). 10.15252/embj.201798049 29472250 PMC5881631

[78] A. E. Conicella , G. H. Zerze , J. Mittal , and N. L. Fawzi , Structure 24(9), 1537–1549 (2016). 10.1016/j.str.2016.07.007 27545621 PMC5014597

[79] B. G. Burnett , J. Andrews , S. Ranganathan , K. H. Fischbeck , and N. A. Di Prospero , Neurobiol. Dis. 30(3), 365–374 (2008). 10.1016/j.nbd.2008.02.007 18417352 PMC2442575

[80] J. Shao , W. J. Welch , N. A. Diprospero , and M. I. Diamond , Mol. Cell Biol. 28(17), 5196–5208 (2008). 10.1128/MCB.00079-08 18573880 PMC2519718

[81] E. J. Alexander , A. Ghanbari Niaki , T. Zhang , J. Sarkar , Y. Liu , R. S. Nirujogi , A. Pandey , S. Myong , and J. Wang , Proc. Natl. Acad. Sci. U. S. A. 115(49), E11485–E11494 (2018). 10.1073/pnas.1811997115 30442662 PMC6298105

[82] R. J. Wheeler , H. O. Lee , I. Poser , A. Pal , T. Doeleman , S. Kishigami , S. Kour , E. N. Anderson , L. Marrone , A. C. Murthy , M. Jahnel , X. Zhang , E. Boczek , A. Fritsch , N. L. Fawzi , J. Sterneckert , U. Pandey , D. C. David , B. G. Davis , A. J. Baldwin , A. Hermann , M. Bickle , S. Alberti , and A. A. Hyman , “Small molecules for modulating protein driven liquid-liquid phase separation in treating neurodegenerative disease,” bioRxiv. 10.1101/721001

[83] H. Deng , K. Gao , and J. Jankovic , Nat. Rev. Neurol. 10(6), 337–348 (2014). 10.1038/nrneurol.2014.78 24840975

[84] S. Schütz , E. R. Nöoldeke , and R. Sprangers , Nucl. Acids Res. 45(11), 6911–6922 (2017). 10.1093/nar/gkx353 28472520 PMC5499654

[85] C. J. Decker , D. Teixeira , and R. Parker , J. Cell Biol. 179(3), 437–449 (2007). 10.1083/jcb.200704147 17984320 PMC2064791

[86] S. H. Ling , C. J. Decker , M. A. Walsh , M. She , R. Parker , and H. Song , Mol. Cell Biol. 28(19), 5965–5976 (2008). 10.1128/MCB.00761-08 18678652 PMC2547010

[87] D. M. Mitrea , J. A. Cika , C. S. Guy , D. Ban , P. R. Banerjee , C. B. Stanley , A. Nourse , A. A. Deniz , and R. W. Kriwacki , Elife 5, e13571 (2016). 10.7554/eLife.13571 26836305 PMC4786410

[88] D. M. Mitrea , J. A. Cika , C. B. Stanley , A. Nourse , P. L. Onuchic , P. R. Banerjee , A. H. Phillips , C. G. Park , A. A. Deniz , and R. W. Kriwacki , Nat. Commun. 9(1), 842 (2018). 10.1038/s41467-018-03255-3 29483575 PMC5827731

[89] N. Kedersha , M. D. Panas , C. A. Achorn , S. Lyons , S. Tisdale , T. Hickman , M. Thomas , J. Lieberman , G. M. McInerney , P. Ivanov , and P. Anderson , J. Cell Biol. 212(7), 845–860 (2016). 10.1083/jcb.201508028 27022092 PMC4810302

[90] S. Markmiller , S. Soltanieh , K. L. Server , R. Mak , W. Jin , M. Y. Fang , E. C. Luo , F. Krach , D. Yang , A. Sen , A. Fulzele , J. M. Wozniak , D. J. Gonzalez , M. W. Kankel , F. B. Gao , E. J. Bennett , E. Lécuyer , and G. W. Yeo , Cell 172(3), 590–604 (2018). 10.1016/j.cell.2017.12.032 29373831 PMC5969999

[91] M. D. Panas , T. Schulte , B. Thaa , T. Sandalova , N. Kedersha , A. Achour , and G. M. McInerney , PLoS Pathog. 11(2), e1004659 (2015). 10.1371/journal.ppat.1004659 25658430 PMC4450067

[92] J. Y. Youn , W. H. Dunham , S. J. Hong , J. D. R. Knight , M. Bashkurov , G. I. Chen , H. Bagci , B. Rathod , G. MacLeod , S. W. M. Eng , S. Angers , Q. Morris , M. Fabian , J. F. Côté , and A. C. Gingras , Mol. Cell. 69(3), 517–532 (2018). 10.1016/j.molcel.2017.12.020 29395067

[93] L. Cirillo , A. Cieren , S. Barbieri , A. Khong , F. Schwager , R. Parker , and M. Gotta , Curr. Biol. 30(4), 698–707 (2020). 10.1016/j.cub.2019.12.020 31956030

[94] C. Huang , Y. Chen , H. Dai , H. Zhang , M. Xie , H. Zhang , F. Chen , X. Kang , X. Bai , and Z. Chen , Cell Death Differ. 27(1), 227–241 (2020). 10.1038/s41418-019-0350-5 31114027 PMC7205891

[95] J. A. Ditlev , L. B. Case , and M. K. Rosen , J. Mol. Biol. 430(23), 4666–4684 (2018). 10.1016/j.jmb.2018.08.003 30099028 PMC6204295

[96] A. S. Lyon , W. B. Peeples , and M. K. Rosen , Nat. Rev. Mol. Cell Biol. 22(3), 215–235 (2021). 33169001 10.1038/s41580-020-00303-z PMC8574987

[97] D. L. J. Lafontaine , J. A. Riback , R. Bascetin , and C. P. Brangwynne , Nat. Rev. Mol. Cell Biol. 22(3), 165–182 (2021). 32873929 10.1038/s41580-020-0272-6

